# Eosinophilic Granulomatosis with Polyangiitis presenting as Vasculitis in the Temporal Artery

**DOI:** 10.31138/mjr.20230727.eg

**Published:** 2023-07-27

**Authors:** Nikolaos Kintrilis

**Affiliations:** Infectious Diseases Unit, 401 General Military Hospital of Athens, Greece

**Keywords:** temporal arteritis, eosinophilic granulomatosis with polyangiitis (EGPA), eosinophilia, vision loss

## Abstract

A 59-year-old man presented to the Emergency Department with vision disturbance, presenting concurrently bronchial asthma and pansinusitis, with complete blood count showing marked eosinophilia (32,420/mL at 79% of white blood cells). Clinical, laboratory, and imaging investigations were unremarkable except for persisting eosinophilia. A histological examination of a biopsied temporal artery showed vasculitis in the temporal artery and concomitant granulomatous inflammation, with lymphocytes, eosinophils, and multinucleated giant cells. Based on the biopsy and a positive anti-myeloperoxidase antibody (anti-MPO/p-ANCA) result, a diagnosis of eosinophilic granulomatosis with polyangiitis (EGPA) was made. The patient was initially treated with methylprednisolone pulses and recovered vision. We consider the present case as EGPA manifesting as temporal arteritis with vision loss and treated it as such, with the patient making a full recovery without further symptomatology occurrences. The current case underlines how ANCA-associated vasculitides can rarely manifest in the form of temporal arteritis.

## INTRODUCTION

Eosinophilic Granulomatosis with Polyangiitis (EGPA) is a multi-organ small to medium vessel anti-neutrophil cytoplasmic antibody (ANCA)-associated vasculitis often presenting in the form of obstructive lung disease and eosinophilia. The current name of the disease replaced the previous term of ‘Churg-Strauss syndrome’ at the 2012 Revised International Chapel Hill consensus conference (CHCC) in an attempt to classify it based more on its histopathologic findings and match the nomenclature terms of other angiitis syndromes (Granulomatosis with Polyangiitis - GPA and Microscopic Polyangiitis - MPA).^1^ Biopsies of affected patients reveal granulomatous inflammation alongside eosinophil infiltrations upon affected tissues, most commonly respiratory system and renal parenchyma.^2,3^ Further reports and studies have since described occurrences of EGPA with the first and/or sole presentation of temporal arteritis.^4,5^

In the current case report, we present a male patient with an EGPA diagnosis based on the 2012 criteria and a sole clinical manifestation of temporal arteritis in the form of vision disturbance, as well as marked eosinophilia.

## CASE PRESENTATION

A 59-year-old retired policeman with a medical history of bronchial asthma since his forties, well controlled under an inhaled combination of budesonide-formoterol, presented to the Emergency Department (ED) of our hospital with complaints of repeated few-minute episodes of transient diplopia and visual loss over the previous few days. Adding to the visual symptoms, he reported a fever of up to 38°C for the preceding few days. He had smoked a half-pack of cigarettes daily up until the asthma diagnosis but had stopped altogether since, drank a glass of red wine daily but consumed no other alcohol, and was of normal body mass index (BMI) at 180cm and 80 kg and generally fit. He had no further complaints other than the visual defects. His vital signs (temperature, blood pressure, heart rate, respiratory rate, and room air oxygen saturation) were normal while physical examination by the internist on duty revealed enlarged, tender temporal arteries, especially on the right side, with equally palpable arterial pulse on both sides. Upon being specifically questioned, the patient denied any headaches, scalp tenderness, jaw claudication and myalgias or arthralgias. A neurological and ophthalmological consultation was asked, the first of which was without findings and the second only revealing beginning signs of left eye cataract. A complete blood count showed marked eosinophilia (32,420/mL at 79% of a total of 41,200/mL white blood cells) and slight anaemia (haemoglobin of 11.1 g/dL and haematocrit of 35.5%). A complete biochemical, coagulation and urinalysis panel were within the normal ranges. C-reactive protein (CRP) was elevated at five times the upper normal limit (25 mg/L, normal laboratory values 0.5–5 mg/L) and erythrocyte sedimentation rate (ESR) also slightly elevated (45 mm/h, normal laboratory values 0–15 mm/h). The patient was admitted to the internal medicine department for further evaluation and diagnosis.

Immunological testing revealed a rheumatoid factor (RF) of 602 U/mL (0–20 U/mL), immunoglobulin (Ig) E of 780 mg/dL (0–100 mg/dL) as well as slight elevations of other immunoglobulins (IgG1, igG2, igG3, IgG4 and total IgG, and a positive myeloperoxidase-anti-neutrophil cytoplasmic antibody (MPO-ANCA, p-ANCA) of 29 U/mL (0–5 U/mL) but negative proteinase-3-anti-neutrophil cytoplasmic antibody (PR3-ANCA, c-ANCA). Hepatitis A, B, C and HIV were excluded. Antinuclear antibodies (ANA), anti-double-stranded DNA (anti-ds DNA), complement 3 and 4 (c3, c4), angiotensin-converting enzyme (ACE), interferon-γ release assay (IGRA) and serum tryptase were negative. A serologic and stool assay were sent for parasites, which were also excluded. A molecular test with reverse transcriptase-polymerase chain reaction (RT-PCR) for detection of the chimeric BCR-ABL gene was negative. Computed tomography (CT) of the chest, brain and abdomen reported pansinusitis and thickening of sinusoidal walls, while a transthoracic echocardiogram (TTE) was normal. A combined gastroscopy and colonoscopy were negative, with obtained biopsies showing no signs of eosinophilic gastroenteritis.

**Figure 1.**
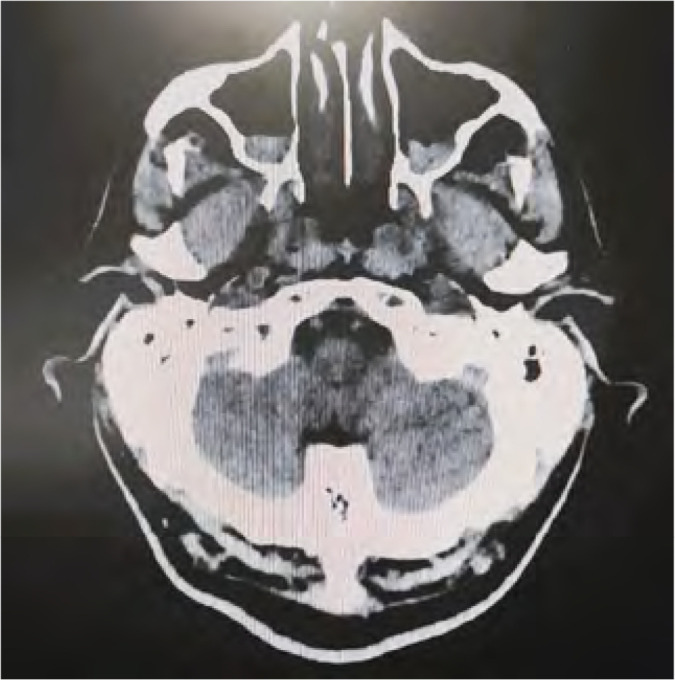
CT of the brain revealed pansinusitis.

**Figure 2.**
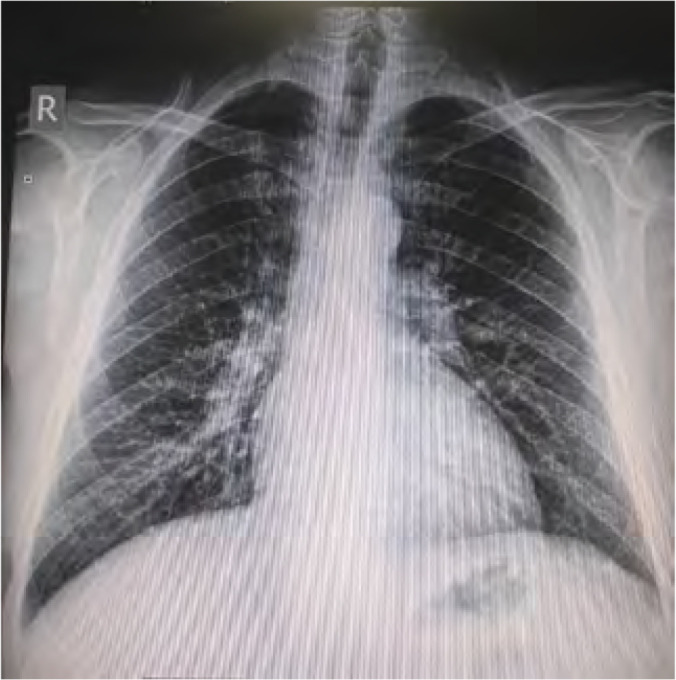
Chest x-ray did not reveal any abnormal findings.

A temporal artery biopsy was performed with a 1.8 cm, 0.1 cm wide part of the right temporal artery being resected, and histological examination was performed, revealing an abundance of eosinophils and lymphocytes in the infected areas of the vessel, and arterial wall thickening. The patient fulfilled the 2022 ACR/European Alliance of Associations for Rheumatology Classification Criteria for EGPA^6^ and was diagnosed accordingly, supporting the notion that temporal artery localised vasculitis can mimic GCA, thus presenting a diagnostic dilemma.^7^ Beginning on the afternoon after his ED attendance, an intravenous methylprednisolone pulse therapy of 1g was administered for 3 days, then changed to prednisolone per os at a dose of 60 mg, tapered slowly over the next months. Ocular symptoms and fever subsided after the application of intravenous pulse therapy and eosinophils dropped rapidly to 9,130/mL at 48.4% of white blood cells after initiation of the corticosteroid therapy (**Table 1**). No further diagnostic tests or therapeutic interventions were performed, and the patient was discharged on the morning of his seventh hospital day.

**Table 1.** Laboratory results before and throughout the admission.

**Parameter**	**Day 0 (ED)**	**Day 1 (pulse therapy start)**	**Day 4 (after 3 days of pulse therapy)**	**Day 7 (discharge)**
White Blood Cells (K/μL)	41.2	27.1	23.0	18.9
Eosinophils (%)	79	58	54	48.4
Eosinophils (K/μL)	32.42	15.61	12.41	9.13
Haemoglobin (g/dL)	11.1	10.5	11.0	10.7
Haematocrit (%)	35.5	33.6	33.3	32.7
ESR (mm/h)	45	48	21	10
CRP (mg/L)	25	23	16	9

## DISCUSSION

We report upon a patient presenting to the ED with complaints of visual disturbances, presenting at the same time asthma and pansinusitis, low-grade fever, and marked eosinophilia, whose workup led to a diagnosis of EGPA. The patient recovered full vision and was discharged after a weeklong hospitalization on a slowly tapered prednisolone regimen, with a swift improvement in his haematological profile. A recent case report identified a total of eleven cases of concomitant EGPA and vasculitis in the temporal artery, seven of which revealed prominent eosinophilia on the temporal artery biopsy, which was also the case in our patient’s biopsied right temporal artery. Four of the cases also had ANCA positivity, another characteristic our patient shared.^8^ Interestingly enough, our patient presented with extremely high eosinophil levels, as well as an abundance of eosinophils in the biopsied temporal artery. This has been the case in only one other patient, of female sex and coincidentally at the same age as our patient, reported in 2004.^9^

In conclusion, we herein reported upon an unusual presentation of EGPA with temporal artery involvement in a middle-aged man, based on the diagnostic criteria. The case adds a cornerstone into the realisation that careful clinical examination of other systems, such as upper and lower respiratory tract, is vital to aid towards the differential diagnosis of temporal arteritis, especially in younger individuals. Only with thorough analysis of clinical complaints, laboratory examinations and histologic findings can a correct diagnosis be reached, helping guide the clinician’s choice of targeted therapy.

## ETHICS AND BIOETHICS

The subject of this case report provided a written informed consent. The Ethics and Bioethics Committee of the 401 General Military Hospital of Athens, Greece was informed of and approved the paper submission.
